# Genetic educational needs and the role of genetics in primary care: a focus group study with multiple perspectives

**DOI:** 10.1186/1471-2296-12-5

**Published:** 2011-02-17

**Authors:** Elisa JF Houwink, Scheltus J van Luijk, Lidewij Henneman, Cees van der Vleuten, Geert Jan Dinant, Martina C Cornel

**Affiliations:** 1 Department of Clinical Genetics, EMGO Institute for Health and Care Research, VU University Medical Center, Amsterdam, The Netherlands; 2 Institute for Medical Education, VU University Medical Center, Amsterdam, The Netherlands; 3 School for Public Health and Primary Care, Department of General Practice, Maastricht University, Maastricht, The Netherlands; 4 Department of Educational Development and Research, Maastricht University, Maastricht, The Netherlands

## Abstract

**Background:**

Available evidence suggests that improvements in genetics education are needed to prepare primary care providers for the impact of ongoing rapid advances in genomics. Postgraduate (physician training) and master (midwifery training) programmes in primary care and public health are failing to meet these perceived educational needs. The aim of this study was to explore the role of genetics in primary care (i.e. family medicine and midwifery care) and the need for education in this area as perceived by primary care providers, patient advocacy groups and clinical genetics professionals.

**Methods:**

Forty-four participants took part in three types of focus groups: mono-disciplinary groups of general practitioners and midwives, respectively and multidisciplinary groups composed of a diverse set of experts. The focus group sessions were audio-taped, transcribed verbatim and analysed using content analysis. Recurrent themes were identified.

**Results:**

Four themes emerged regarding the educational needs and the role of genetics in primary care: (1) genetics knowledge, (2) family history, (3) ethical dilemmas and psychosocial effects in relation to genetics and (4) insight into the organisation and role of clinical genetics services. These themes reflect a shift in the role of genetics in primary care with implications for education. Although all focus group participants acknowledged the importance of genetics education, general practitioners felt this need more urgently than midwives and more strongly emphasized their perceived knowledge deficiencies.

**Conclusion:**

The responsibilities of primary care providers with regard to genetics require further study. The results of this study will help to develop effective genetics education strategies to improve primary care providers' competencies in this area. More research into the educational priorities in genetics is needed to design courses that are suitable for postgraduate and master programmes for general practitioners and midwives.

## Background

In the age of genomics, the genetics of common chronic disorders, pharmacogenetics and large-scale applications in screening are becoming increasingly important. Primary care providers (e.g. general practitioners and midwives) will have to discuss these issues with their patients, who are becoming increasingly aware of genetic contributions to disease and also have high expectations of genetic testing [1]. Consequently, primary care providers need to be educated to meet the needs of their patients that are created by rapid advances in genomics [2]. Genetics literacy among primary care providers needs to be improved to enable their participation in the debate on the hopes and hypes of genomic medicine and to distinguish between useful and useless practical applications in health care [3]. Currently, genetics and genomics are rather underrepresented in postgraduate (physician) training programmes in general practice (here, the terms *general practice *and *family medicine *(commonly used terms in the Dutch health care system) are considered synonymous to the more commonly used term *family practice *in the U.S. healthcare system) as well as in master programmes in midwifery and public health [4,5]. It is widely recognised that medical professionals and medical students should be educated about genetics [2,5-7].

Research into the perspectives of general practitioners and midwives on the educational priorities and attitudes in relation to genetics [8-16] revealed a need for genetics education for primary care providers in areas like psychosocial issues and screening, assessment of the risk of genetic malformations and basic genetics. However, the educational needs of primary care providers and their views on the role of genetics in family practice are still under investigation, and international efforts to translate these needs into education programmes are still in their early stages [17,18].

Primary care providers have a unique role in the Dutch health care system and general practitioners are easily accessible to all patients for any complaint, request or question. Midwives provide obstetric and perinatal care and give advice and guidance to patients on pregnancy and childbirth. Genetics could have an effect on daily primary care practice if basic and clinical science advances in genomics of common chronic diseases in practice and midwifery care are successfully translated [2,3,19]. Changes will only be effective, however, if they fit well into practice routines. It is therefore important to understand how these key professional groups conceive of their responsibilities and experiences in relation to genetics. For the implementation of genetics training in daily practice to be successful, it is important to identify factors that can enhance or inhibit effective genetic primary care.

Up till now, most studies of educational needs concerning genetics have been limited to the perspectives of target groups [1,4-14]. Professional training needs can be derived from those studies and from challenges posed by new applications as foreseen by experts. This study explored the views of general practitioners, midwives, patient advocacy groups and others involved in genetics in health care and education regarding their need for genetics education and the role of genetics in primary care. This information was collected to help develop effective genetics education and training as well as effective integration of genetics in primary care.

## Method

### Design

We used a qualitative study design with focus groups because this enables the exploration of the meaning and significance of the role of genetics and the need for education in that area as perceived by different stakeholders. A discussion with members of the research team and primary care providers revealed that general practitioners and midwives were the primary care providers most likely to be confronted with issues of genetics and genomics in their practices.

### Participants

We used purposive sampling to recruit specific groups of professionals for focus group interviews in order to obtain rich, relevant and diverse data.

The participants were expected to provide complete and possibly complementary perspectives on genetics in primary care practice and education. Potential participants were named by key persons and network contacts at academic departments of general practice and the midwifery academies at Amsterdam and Maastricht, the Netherlands.

We convened three types of focus groups, (1) two groups of general practitioners, (2) two groups of midwives, and (3) three multidisciplinary groups composed of clinical genetics professionals (clinical geneticists or genetic counsellors), primary care educators, and representatives of patient advocacy groups. The participants in the multidisciplinary groups were considered experts who were expected to have a broad view of the role of genetics in primary care and the need for genetics education i.e. what is needed, what works and what does not work.

For each focus group, ten to fifteen professionals from one region were invited by email or telephone. Those who responded positively received an invitational letter, informing them that the purpose of the study was to explore their perceptions of the role of genetics and genomics in primary care and the related education needs. The term 'genetics' was commonly used during the focus group discussions, and the term 'genomics' was used to denote a broad definition (e.g. common complex disorders and rapid technological developments).

### Focus groups

Additional file 1 provides an overview of the participants. The interviews lasted approximately two hours and were held between March and August 2009. The discussions were facilitated by an independent and experienced moderator (SL), who encouraged the participants to participate actively and to openly state their viewpoints and engage in discussion. An assistant (IH) took notes and all the sessions were attended by one observer (LH). Participation was voluntary and participants received €100 plus travel expenses.

The study was approved by the Medical Ethics Committee of VU University Medical Center, Amsterdam and Maastricht University. All participants gave informed consent at the start of their focus group session.

### Interview guide

An interview guide with open-ended questions was developed to ensure coverage of the major topics (Additional file 2). The moderator opened each focus group interview with an introduction and a round-robin open question: "What are your experiences with genetics/genomics in primary care?" Further probing by the moderator was rarely required, since each group spontaneously talked about genetics education needs and the role of genetics in primary care. When needed, asking for clarification of the answers was sufficient to elicit ample additional information.

### Data analysis

The focus groups were audio recorded and transcribed verbatim. Member checking entailed sending a summary of the sessions to all participants and inviting their comments, which were then incorporated in the transcripts. Using Atlas.ti5.2 for data analysis, two of the researchers (IH and LH) independently coded the themes that emerged from the transcripts. They compared their coding for reliability and reached consensus on differences through discussion. Through a process of discussion and deliberation, connections between the codes were identified and categories and themes developed. The transcripts were read repeatedly to check the accuracy and completeness of the themes and subthemes. In the results section, representative quotes from the focus groups, translated from the Dutch, are presented to illustrate the themes.

## Results

All participants acknowledged the importance of genetics education for primary care providers. A need for education was expressed more urgently by the general practitioners than by the midwives, while members of the multidisciplinary group generally indicated that both groups were deficient in genetics knowledge and skills. The extent and focus of the discussions differed between the general practitioners and the midwives, because the general practitioners saw themselves as generalists while perinatal care was the primary focus for the midwives. This difference was reflected in their priorities for education.

Four distinct themes emerged: (1) the need for genetics knowledge, (2) taking a family history, (3) ethical dilemmas and psychosocial effects related to genetics, and (4) insight into the organisation and role of clinical genetics services (Additional file 3).

1. The need for genetics knowledge

General practitioners perceived deficiencies in their basic understanding of genetics, since they had never been taught this or because their knowledge had faded. They experienced their lack of knowledge as a barrier to the use of genetics in diagnosis, treatment and in consulting clinical geneticists, and they expressed a strong need for this knowledge, as reflected in the words of one general practitioner (male, 37 years):

"I think it's essential to know the basics [of genetics].... You should know what a gene is, that there are deviant genes and that genes can be turned on and off... These are basics that clarify genetics and make it understandable so that it can be translated to patients in relation to diagnosis or treatment or to advise them to refrain from something, smoking, for example."

Other participants acknowledged this need and argued that general practitioners should have more knowledge in order to provide general genetic information. Primary care providers wondered how much they really needed to know about such a complex field. All groups shared the view that primary care providers cannot be expected to know everything. Since many genetic diseases are rarely seen in primary care, there is no urgent need for them to be included in training programmes.

General practitioners appeared to be generally aware of their lack of knowledge, which made them ill equipped to identify genetic problems in their patients. In response to the question about experiences with genetics/genomics, one general practitioner (male, 52 years) said:

"I desperately hope the midwife or obstetrician will think of [prenatal diagnosis for women of advanced maternal age], because I don't always think of bringing it up. Also, I tend to think the specialist will consider all the genetic aspects of a clinical problem, but this often is not the case."

Midwives did not perceive a lack of genetics knowledge and said the midwifery master programme provided sufficient education on this topic. Multidisciplinary group members, however, said that both general practitioners and midwives needed education to increase their knowledge, as they observed a lack of knowledge in both groups.

There was a discrepancy in the content of the required knowledge as perceived by the participants. General practitioners mentioned a need for knowledge about prevention of common genetic diseases, whereas the midwives were primarily interested in prevention of perinatal diseases. To meet these educational needs, general practitioners and multidisciplinary experts believed that genetics education should address family history and inheritance patterns.

Both general practitioners and midwives were interested to know where they could find more genetic information. There appeared to be a general need for easily accessible sources of information such as web-based education or websites with short and easy to understand information that could be applied in daily practice.

2. Taking a family history

Primary care providers believed that taking a family history was extremely important as it allows for familial risk stratification and identification of hereditary conditions. For midwives the importance of family history was limited to perinatal disease. The need to increase awareness of familial diseases in primary care was discussed by a midwifery educator (female, medical doctor, 56 years):

"We agree on when to think of some familial diseases. I mean, it's clear when speaking of colon carcinoma or breast cancer that you realise [as a general practitioner] that it can be inherited. There should be a clinical guideline to help one decide when to consult a clinical geneticist for further diagnosis. Today, more general practitioners should realise this [colon carcinoma or breast cancer] could be familial."

An educator, allied to a postgraduate general practice programme, thought that taking a family history was well covered during postgraduate training, but the general practitioners were not quite so sure. Although family history was part of some consultations, most general practitioners said that family history and pedigree drawing were not part of their daily routine. The midwives said that family history was something they did every day, but they lacked the skills for pedigree drawing. They thought this was not important, however, because they did not do it often enough to maintain this skill. They thought the main thing was to be able to recognise high-risk factors in a family history

"Midwives generally lack sufficient knowledge to draw a pedigree. I think the same applies for general practitioners, but it is more important for them [general practitioners] to detect high-risk family history criteria. How and when is something important? The signalling function, that is important and it should be taught. Counselling is important for both general practitioners and midwives. But above all it is important to be clear on when something is important, how important it is for you, what you want to do about it, and once this is clear you can take the next step" (a female GP and midwifery educator, 39 years).

General practitioners were uncertain about recording information from family history in the electronic patient record. Participants said that measures should be taken to improve the electronic patient record to include information from family history.

3. Ethical dilemmas and psychosocial effects related to genetics

Most primary care providers expressed concern about the surge of genetic testing which confronted them with ethical dilemmas and more profound psychosocial effects of genetics in their daily practice. One participant said he was faced with "an increasing amount of vague, worrying and inexplicable genetic information". He referred to information about genetic risks provided to consumers by commercially available Personal Genome Services. Primary care providers felt unqualified to deal with these issues and thought that genetic ethical dilemmas should be part of genetics education.

General practitioners also wondered whether it was beneficial to their patients and themselves to know everything about a patient's genetic background. They voiced concern about the possibility of unauthorised dissemination of genetic information and related privacy issues if it were to become obligatory for them to take a family history and record the information derived from it. They asked: "Who should you inform about genetic information and who should you not inform if you want to keep your patient's best interest at heart, for example when a patient wants to take out life insurance or needs a mortgage to buy a new house?"

Midwives thought that developments in genetics were moving at a very rapid pace, giving rise to feelings of insecurity both in midwives and their patients. They discussed whether following the protocol for perinatal screening might be inappropriate and therefore not uniformly applicable. They preferred to adapt the application of genetic protocols to individual patients, because test results could have important genetic and personal consequences. One midwife, (female, 40 years) explained:

"Surely because this child is already in the uterus, the basic question is rather what do you really want to know? Because what are you going to do with this [genetic] information?"

Clear guidelines as to when a general practitioner should be proactive and bring up the subject of familial disease to patients and their families were non-existent but considered necessary. General practitioners were unclear about how to guide patients in their decisions around prenatal and genetic testing. As a result they perceived non-directive counselling as difficult because it was influenced by their personal opinions and sense of urgency. One general practitioner (male, 52 years) explained:

"I used to discuss prenatal screening with patients at first when it was a hot item. Everybody wanted the triple test, but this seems to change when you explain that it gives a probability that doesn't offer any certainty and things can also happen after the baby is born or you cannot always see things on the outside and people then pull back automatically. I try to counsel nondirectively, because it promotes shared responsibility. I like to hold on to this nice shared responsibility, because it doesn't make me feel that I am solely accountable."

General practitioners and midwives alike mentioned consanguinity as a complex issue to discuss with patients and they raised the problem of how to deal with a potentially increased risk of congenital disease. One general practitioner (female, 52 years) said:

"When cousins get married, they are blissfully happy when presenting this news to their doctor. As a general practitioner I find it complicated, when there is a disease in the family, to confront these people with this problem in today's society."

Inter-cultural differences were considered a source of difficulties in discussing prenatal or preconceptional screening. Midwives sensed urgency early on in pregnancy when recommending this type of screening to members of ethnic minorities in order to prevent congenital disease. This feeling of urgency was sometimes enhanced by language barriers and time constraints.

4. Insight into the organisation and role of clinical genetics services

General practitioners expressed a need for education with regard to indications for referral to clinical genetics services. Some general practitioners preferred to first gather information from other sources (such as online websites) before turning to a clinical geneticist. Other general practitioners said it was better to refer than to do it all yourself. Midwives, on the other hand, said it was easy for them to consult clinical geneticists, whom they regularly telephoned for advice.

Some general practitioners and midwives said it was not clear to them what clinical geneticists do and what the clinical trajectory would be once a patient was referred to such a service. General practitioners mentioned their lack of familiarity with this type of service as a cause of inappropriate consultation strategies, which could result in untimely referrals. Geneticists argued that general practitioners should consult them more often, and that this should be stimulated by education.

"The relation between primary and secondary care is sometimes difficult. Once the clinical geneticist has diagnosed a certain genetic disease, which means more specialised information, the patient's family members find themselves in a pickle together with the GP and then the whole process (of consulting) starts all over again. [...] I hope education of primary care providers will result in accessible consultation services. Because I don't think the general practitioner should know everything about everything ... but they should at least know that help is easily available." (Clinical geneticist, female, 45 years).

### The role of genetics in primary care

The role of genetics in primary care was perceived to be unduly limited as a result of care providers' inadequate genetics knowledge and skills. Although care providers might show some interest in improving their knowledge, representatives of patient advocacy groups indicated that primary care providers were "not sufficiently proactive" in this area. They perceived an urgent need for inclusion of genetics in primary care guidelines in order to make genetics a "hot item". General practitioners and midwives said they were unsure about their responsibilities in relation to genetics, perhaps because they lacked insight into the genetic background of diseases and its possible consequences. A representative of a patient advocacy group (male, 59 years) said:

"No knowledge (of genetics) and no interest (in genetics). It's not a hot item. It seems as if general practitioners are not interested in identifying patients with familial hypercholesterolemia (FH). We have an excellent screening programme for this in the Netherlands, which threatens to go under because too few index patients are put forward by general practitioners. General practitioners do not alert patients that they might have FH when there is a positive family history and high cholesterol levels, and patients are not advised to take part in a brief screening programme [...]. General practitioners often say they don't have the time or they're not interested or they see no benefit."

Primary care providers noticed a change in their experiences with and views of the role of genetics in primary care, which led to an increased need for basic knowledge of genetics and family history taking. General practitioners felt their current knowledge was insufficient to meet these needs. Participants also said they noticed an increase in patients' questions about genetic issues. They perceived a change in the responsibilities of primary care providers that prompted increased attention for genetics. They also saw an urgent need for a description of the responsibilities of different disciplines in relation to genetic issues. A clinical geneticist (female, 40 years) put it as follows:

"When I think of genetics I think of monogenetic disorders, but of course disorders (seen in primary care) are often complex disorders or multigene or gene environment interaction disorders. Of course, clinical genetics cannot deal with all those problems, it's simply impossible. [...] I think it is the task of the general practitioner, but I find this difficult... is it really the task of the general practitioner to deal with such complicated problems?"

Overall, participants were positive about the changed role of genetics in primary care. They said this change emphasised their role as an easily accessible source of information. However, there was also some criticism. General practitioners saw even more work coming their way, which caused some concern. Taking a family history, non-directive counselling, and unfamiliarity with recording information from family history in their electronic patient record were said to take up a great deal of time.

General practitioners indicated that they were aware of rapid developments in genetics and the subsequent lag in its application in primary care. They regarded this as important and pointed to two aspects of this change. Firstly, they said it was urgent for limits to be set in relation to required genetics-related knowledge and responsibilities in primary care. Secondly, education should include the clinical application of genetic developments and ways to communicate genetic information. A midwifery educator (female, medical doctor, 56 years) clarified these aspects:

"Highly educated people develop national genetics guidelines. Even the ethical issues involved in these problems and how to deal with them are prescribed. In primary care you are in close contact with patients and it can be difficult sometimes to apply theory-based guidelines in a way that can be understood by patients, it is difficult to do this appropriately."

### Suggestions for strategies for effective genetic education

At the end of each focus group interview, the participants were asked to briefly consider effective strategies for teaching genetics in primary care education (Additional file 4).

The following general considerations emerged: programmes should be relevant to primary care practice, participants in the multidisciplinary group emphasised the importance of assuring the quality of educational strategies and suggested that programmes should range in duration from brief sessions to ten-day programmes. Finally, strategies should be added to existing programmes or could be integrated with other topics, such as cardiovascular risk management or familial breast cancer as examples of common diseases.

## Discussion

The results of this study indicate that Dutch primary care providers need, and would welcome, more extensive education in genetics. Four major themes emerged in relation to the role of genetics in primary care and the related educational needs: lack of basic knowledge, need for education on family history taking and the potential clinical consequences, ethical dilemmas and psychosocial effects related to genetics and insight into the organisation of regional genetics services and the referral system. There was general agreement that increased genetics knowledge and family history taking by primary care providers would require a better understanding of the organisation of genetics services in order to promote more appropriate and timely referrals. In summary, the results point to a need for courses in genetics for master programmes in midwifery and postgraduate programmes in family medicine.

A similar need for genetics education in primary care was also found in other studies. The identified needs are in line with the learning outcomes and core competencies in genetics proposed by genetics experts for non-genetic health care professionals [17,18]. Since there is little published research on the extent to which the need for genetics education matches the core competencies, we used a qualitative approach to explore the views of the target group. In this way we gained insight into the educational needs of this group with regard to genetics; general practitioners indicated that a paucity of knowledge can lead to poor recognition of and unresponsivess to genetic problems in daily patient care.

The results of this study are in line with some studies and differ from others with regard to the need for increased genetics knowledge among midwives [8,13] and general practitioners [5,10,12,14,16,19,20]. The midwives in our study seemed more confident of their basic knowledge and did not perceive as strong a need to adapt existing educational programmes as was expressed by midwives in studies by Benjamin *et al*. and Metcalfe *et al*. [8,13]. This difference may be due to differences between master programmes in midwifery or between health care systems.

Our results support the outcomes of the afore mentioned studies regarding deficiency in skills (e.g. taking a family history, referral to appropriate regional genetics services and non-directive counselling) [8,13]. It may be problematic for primary care providers to take appropriate steps in response to the perceived shift in the importance of genetics in primary care, such as taking enough time to discuss the family history or non-directive counselling. Another step to take would be to improve the electronic patient record in order to achieve accurate documentation of family history information.

Martin and Wilikofsky reported on general practitioners' perceptions of their role in genetic counselling and their unwillingness to accept this role due to time and organisational constraints [21]. Representatives of patient advocacy groups and genetic counsellors in our study emphasised the need to increase acceptance of the importance of genetics and genetic counselling in primary care. The responsibility, on the part of the patient or the doctor, to report data from the family history remains a topic of debate, however, even though the importance is clear and primary care seems well suited to include this role in daily practice routines[22]. Perhaps a joint effort by all stakeholders would be realistic and useful.

General practitioners and other participants in our focus groups recognised the important role of genetics in primary care. This is in contrast to a study conducted by Fetters *et al*. in 1999 [12], which found general practitioners reluctant to invest in self-education in genetics, because they felt genetic problems were not clinically relevant. Our study suggests that today's primary care providers are aware of a progressive impact of genetics on primary care and therefore increasingly conscious of what they don't know. They recognise the need for attention to genetics in educational programmes. Perhaps this is a reflection of family medicine finally becoming aware that genetics and genomics are an integral part of primary care.

Clinicians were seen to be uncomfortable in applying genetics in their daily practice, which resulted in difficulties in referring adult patients for genetic counselling[7]. Our study showed similar results. Some general practitioners were reluctant to consult a clinical geneticist, whereas midwives seemed to be more comfortable with this. Representatives from patient organisations were also aware of this barrier and urged more genetic education for primary care providers, general practitioners in particular. Taylor *et al*. also suggested that insurance coverage of genetic consultation can be a problem. There is currently a paucity of published research on the clinical value of genetic evaluation in primary care [23-25]. Genetic counselling could be of greater value and might be integrated in periodical check-ups more often if its results had greater practical applicability.

The educational strategies suggested by general practitioners and midwives in this study appear to be supported by Gaff *et al*. [26], who concluded that "Program logic, adult learning theory, and evaluation theory together provide a useful and relevant theoretic framework for the development of genetics education programs for health professionals."

### Limitations

The use of focus groups has engaged primary care providers of a potential genetics education programme in the Netherlands. A variation in concepts is possible, because it is unknown how far the themes reach in their contribution and interaction in real practice. The aim of this study was intended to yield results regarding the participants' particular views on knowledge, skills and attitudes in relation to genetics education in primary care. Apart from homogenous groups of general practitioners and midwives, we included participants from a variety of backgrounds to obtain input on broader and future developments in genetics in primary care. However, it remains to be investigated if the results have relevance beyond the Dutch health care system, since the nature of the sample was drawn from this particular health care system.

Together with previously published studies on various aspects of genetics in primary care education, our study offers a broad perspective on genetics education. We believe this information can be used to develop genetics education programmes in the near future. The inclusion of multidisciplinary focus groups which could provide meta views can be considered a strength but also a weakness of this study because of the unequal representation of different fields of expertise in these groups. Another limitation is that purposive sampling can result in self-selection, which can introduce bias. Our study revealed four major themes concerning the role of genetics in primary care. In order to ensure that our picture is complete and usable for educational purposes, and possibly for policy makers as well, consensus has to be sought, for example by means of a Delphi procedure.

## Conclusions

The results of this study suggest that postgraduate training in primary care could be enhanced by incorporating additional training in basic clinical genetics. For midwives and general practitioners there should be more emphasis on counselling using strategies that are clinically feasible and on ethical issues relating to genetic conditions. Insight into the organisation of regional genetics services and the referral system should be enhanced to promote interdisciplinary collaboration. There is an urgent need for a clear description of responsibilities and guidelines to enable effective use of developments in genetics in primary care. Especially descriptions of the genetic responsibilities of primary care providers and their specific role in this area will have to be addressed by future research. Useful and effective application of genetics knowledge can only become a reality when genetics education is improved.

## Competing interests

The authors declare that they have no competing interests.

## Authors' contributions

All authors made a substantial contribution to the concept and design of the study. IH organised and attended all the focus groups, wrote the summaries for member checking, carried out the analysis together with LH and wrote the first draft of the paper. SJvL was moderator of the focus groups. LH attended the focus groups to take notes and observe. SJvL and LH participated in the design of the study and the development of the focus group scenario. CvdV, GJD and MC were involved in coordination of the study and design.

## Pre-publication history

The pre-publication history for this paper can be accessed here:

http://www.biomedcentral.com/1471-2296/12/5/prepub

## Supplementary Material

Additional file 1 **Table 1 Characteristics of the participants in the focus groups**. Click here for file

Additional file 2 **Table 2 Interview guide for the focus group discussions**. Click here for file

Additional file 3 **Table 3 Themes and subthemes identified regarding the genetic educational needs of general practitioners and midwives**. Click here for file

Additional file 4 **Table 4 Suggested strategies, including details, for effective ways to incorporate genetics into primary care education**. CME continuing medical education. Click here for file
