# Supplementary material for: Genetic educational needs and the role of genetics in primary care: a focus group study with multiple perspectives
Source: BMC Fam Pract. 2011 Feb 17;12:5. doi: 10.1186/1471-2296-12-5 (PMC3053218; doi:10.1186/1471-2296-12-5)
Supplement: Additional file 1 — Table 1 Characteristics of the participants in the focus groups. [file 1471-2296-12-5-S1.DOC]

| **Type of focus group** | **N** | **Female**  **(N)** | **Mean age in years (SD)** | **Mean work experience in years (SD)** | **Professional background** |
| --- | --- | --- | --- | --- | --- |
| General practitioner group 1 | 7 | 3 | 40.6 (8.1) | 11.6 (7.8) | general practitioner |
| General practitioner group 2 | 6 | 3 | 49.3 (10.2) | 19.2 (12.3) | general practitioner |
|  |  |  |  |  |  |
| **General practitioner group total** | **13** | **6** | **45 (8.7)** | **15.4 (9.7)** |  |
|  |  |  |  |  |  |
| Midwife group 1 | 8 | 8 | 39.3 (6.1) | 13.5 (6.2) | midwives |
| Midwife group 2 | 6 | 6 | 32.2 (7.9) | 7.8 (6.6) | midwives |
|  |  |  |  |  |  |
| **Midwife group total** | **14** | **14** | **35.7 (6.7)** | **10.6 (6.1)** |  |
|  |  |  |  |  |  |
| Multidisciplinary  group 1 | 6 | 5 | 52.2 (8.3) |  | 3 midwives-midwifery teachers, 1 medical doctor-midwifery teacher, 1 researcher,  1 medical psychologist |
| Multidisciplinary  group 2 | 7 | 5 | 49.7 (6.0) |  | 2 patient organisation representatives,  2 medical doctors, 1 midwife-midwifery teacher, 1 policy advisor, 1 clinical geneticist |
| Multidisciplinary  group 3 | 4 | 3 | 47.8 (14.6) |  | 2 patient organisation representatives,  1 genetic counsellor, 1 policy advisor |
|  |  |  |  |  |  |
| **Multidisciplinary**  **group total** | **17** | **13** | **49.9 (8.7)** |  |  |

**Additional file 1 Table 1 Characteristics of the participants in the focus groups**
