# Supplementary material for: Genetic educational needs and the role of genetics in primary care: a focus group study with multiple perspectives
Source: BMC Fam Pract. 2011 Feb 17;12:5. doi: 10.1186/1471-2296-12-5 (PMC3053218; doi:10.1186/1471-2296-12-5)
Supplement: Additional file 2 — Table 2 Interview guide for the focus group discussions. [file 1471-2296-12-5-S2.DOC]

| **Genetics in primary care; assessment of need for inclusion of genetics in primary care education** |
| --- |
| What comes to mind when you think of genetics in primary care? |
| Do you think primary care workers are capable of answering questions about genetics? Why (not)? |
|  |
| **Genetics in primary care; assessment of the role of genetics in primary care** |
| In your opinion, what is the role of genetics/genomics in primary care today and what will it be in the near future? |
| What is your opinion about the genetic knowledge and skills currently available to primary care providers to fulfil this role? |
| In your opinion, what are the most important genetic topics for the education of general practitioners and midwives? |

**Additional file 2 Table 2 Interview guide for the focus group discussions**
