# Supplementary material for: Genetic educational needs and the role of genetics in primary care: a focus group study with multiple perspectives
Source: BMC Fam Pract. 2011 Feb 17;12:5. doi: 10.1186/1471-2296-12-5 (PMC3053218; doi:10.1186/1471-2296-12-5)
Supplement: Additional file 3 — Table 3 Themes and subthemes identified regarding the genetic educational needs of general practitioners and midwives. [file 1471-2296-12-5-S3.DOC]

**Additional file 3 Table 3 Themes and subthemes identified regarding the genetic educational needs of general practitioners and midwives**

| **Themes** | **General practitioners** | **Midwives** |
| --- | --- | --- |
| **1. The need for genetic knowledge** | Perceived need for genetic knowledge  Aware of lack of knowledge  Cannot know everything  Knowledge should be aimed at prevention of common diseases  Want to know where to find genetic information | No perceived need of genetic knowledge  Not aware of lack of knowledge  Knowledge should be aimed at prevention of perinatal diseases  Want to know where to find genetic information |
| **2. Taking a family history** | Taking a family history and pedigree drawing is not routine  Systematically taking a family history could be improved  Registering family history is complicated | Taking a family history is routine  Pedigree drawing is not routine; infrequently carried out and therefore skill not maintainable |
| **3. Genetic ethical dilemmas and psychosocial effects** | Increasingly confronted with genetic dilemmas and psychosocial issues  Privacy issues and genetics complex  Questions on what should be genetically identified and what should not  Perceived difficulty of non-directiveness in consultations  Discussing consanguinity is difficult | Genetic developments in perinatal screening are rapid; raises more ethical questions  Discussing consanguinity is difficult  Discussing preconceptional and prenatal screening is difficult due to inter-cultural differences |
| **4. Insight into the organisation and role of clinical genetics services** | Do not know when to refer to clinical genetics services  Prefer to sort things out by themselves first  Little insight into the organisation of clinical genetics centres and clinical trajectory | Consult clinical geneticists easily  Little insight into the organisation of clinical genetics centres and clinical trajectory |
