# Supplementary material for: Genetic educational needs and the role of genetics in primary care: a focus group study with multiple perspectives
Source: BMC Fam Pract. 2011 Feb 17;12:5. doi: 10.1186/1471-2296-12-5 (PMC3053218; doi:10.1186/1471-2296-12-5)
Supplement: Additional file 4 — Table 4 Suggested strategies, including details, for effective ways to incorporate genetics into primary care education. CME continuing medical education. [file 1471-2296-12-5-S4.DOC]

***Additional file 4 Table 4 Suggested strategies, including details, for effective ways to incorporate genetics into primary care education***

| **Strategies** | **Details** |
| --- | --- |
| A short internship in the clinical genetics department to familiarise students with the specialty | Suggested by clinical genetics professionals, primary care educators and younger general practitioners |
| E-learning | Younger participants were in favour |
| Lectures | Preferred by older participants |
| New guidelines which should be easily accessible on a website | A decision tree should be attached to help GPs and midwives with busy schedules to quickly find genetic information about how, when and to whom to refer. |
| Workshops | Learning by discussing clinical cases with colleagues and attending clinical geneticists, paediatricians or other specialists depending on the topic under discussion.  Basic genetic knowledge, skills, such as drawing a pedigree, and attitude, through discussing medical ethical topics, could be included. |
| Continuing Medical Education | CME genetics sessions repeated yearly and accredited with CME credits to promote attendance. |

CME = Continuing Medical Education
